# Unraveling the difference in flavor characteristics of *Huangjiu* fermented with different rice varieties using dynamic sensory evaluation and comprehensive two-dimensional gas chromatography–quadrupole mass spectrometry

**DOI:** 10.3389/fnut.2023.1160954

**Published:** 2023-06-22

**Authors:** Haiyan Yu, Qiaowei Li, Wei Guo, Lianzhong Ai, Chen Chen, Huaixiang Tian

**Affiliations:** ^1^Department of Food Science and Technology, Shanghai Institute of Technology, Shanghai, China; ^2^School of Medical Instrument and Food Engineering, University of Shanghai for Science and Technology, Shanghai, China

**Keywords:** *Huangjiu* (Chinese rice wine), rice varieties, flavor characteristics, dynamic sensory evaluation, comprehensive two-dimensional gas chromatography–mass spectrometry

## Abstract

To investigate the specific differences in flavor characteristics of *Huangjiu* fermented with different rice varieties, dynamic sensory evaluation, comprehensive two-dimensional gas chromatography-quadrupole mass spectrometry (GC × GC–qMS) and multivariate statistical analysis were employed. Dynamic sensory evaluation methods including temporal dominance of sensations (TDS) and temporal check all that apply (TCATA) were applied to explore the differences and variations in sensory attributes. The sensory results showed that the intensity of astringency and post-bitterness in the *Huangjiu* fermented with glutinous rice was weaker while ester and alcoholic aroma were more prominent than the one fermented with japonica rice. The results of free amino acids and aroma compounds analysis indicated that the amino acids were mainly sweet and bitter amino acids, and some key aroma compounds were predominant in the *Huangjiu* fermented with glutinous rice, such as ethyl butyrate (OAV: 38–59), 3–methylthiopropionaldehyde (OAV: 47–96), ethyl caprylate (OAV: 30–38), while nonanal, phenyl acetaldehyde and vanillin contributed significantly to the *Huangjiu* fermented with japonica rice. The multivariate statistical analysis further confirmed that 17 compounds (VIP > 1 and *p* < 0.05) could be supposed to be the key compouns that cause significant flavor differences in *Huangjiu* samples fermented with different brewing rice. Moreover, partial least-squares analysis revealed that most compounds (ethyl butyrate, 3-penten-2-one, isoamyl acetate, and so on) correlated with ester and alcoholic aroma. The results could provide basic data and theoretical basis for the selection of raw materials in *Huangjiu*.

## 1. Introduction

*Huangjiu* (Chinese rice wine), with a strong ester aroma and taste of rich and full-bodied, is one of the oldest fermented alcoholic beverages in the world and popular among consumers in East Asia (1). It is typically fermented with unique raw materials (brewing rice, brewing water and saccharification starter). The main rice varieties used in *Huangjiu* are glutinous rice and japonica rice, and their differences in amylopectin, protein and fat will affect the flavor quality of *Huangjiu*. The flavors of different *Huangjiu* brewed from indica glutinous rice, japonica glutinous rice and daily rice were compared and analyzed, and found that the content of volatile flavor compounds such as ethyl esters in *Huangjiu* brewed from japonica glutinous rice was significantly higher than that of the other two kinds of rice (2). In addition, there were significant differences in the concentrations of sweet, bitter, fresh and astringent amino acids in Chinese rice wine brewed from different rice varieties (3). The old saying that “rice is the flesh of Shaoxing *Huangjiu*” intuitively further reveals the importance of brewing rice to *Huangjiu*.

The unique raw materials and fermentation process endowed *Huangjiu* in Shaoxing region with the taste characteristics of fresh, sweet, mellow, and refreshing (4). At present, the taste evaluation of *Huangjiu* was mainly based on descriptive sensory analysis, which focused on static judgment of the sensory attributes or intensity of the sample within a given time (5), ignoring that the perception of sensory attributes may change with the residence time in the mouth (6). Dynamic sensory evaluation techniques represented by temporal dominance of sensation (TDS) and temporal check-all-that-apply (TCATA) can clearly captured the dynamic changes of various taste attributes and simultaneously compared and analyzed multiple taste attributes. Therefore, dynamic sensory evaluation techniques have been applied in the taste evaluation of beverages and wines (7, 8). Comparative analysis on the differences in physicochemical indexes and flavor characteristics of *Huangjiu* brewed with different rice varieties were studied (9, 10). Descriptive analysis is the main sensory evaluation method to reveal the flavor characteristics of *Huangjiu*. To our knowledge, there is no research on the flavor characteristics of *Huangjiu* brewed with different rice using dynamic sensory evaluation method.

Alcohols, esters, acids, aldehydes and ketones are the main volatile aroma compounds of *Huangjiu*, which has been extensively studied by researchers using gas chromatography–mass spectrometry (11, 12). However, due to the low resolution, sensitivity and insufficient separation ability of one-dimensional GC–MS (13), the actual flavor components were more abundant than those detected. Comprehensive two-dimensional gas chromatography (GC × GC) can overcome the defects with the advantages of high selectivity and sensitivity, large peak capacity and higher recognition ability. Two-dimensional gas chromatography is widely combined with rapid scanning quadrupole mass spectrometry (qMS) and time-of-flight mass spectrometry (TOF-MS) to analyze the aroma compounds in wine (14, 15). In our previous study, GC × GC-qMS was used to analyze the aroma characteristics of *Huangjiu* in Shaoxing region with different vintage (16) and different brewing water (17). The conclusions of these researches showed that the coverage of aroma compounds of GC × GC-qMS was much higher than that of GC–MS. However, current research mainly applied GC–MS to compare and analyze the aroma compounds of *Huangjiu* brewed by different rice varieties, the identification of aroma component involving GC × GC-qMS analysis are still insufficient.

The objectives of this study are (1) to evaluate the differences in taste and aroma characteristics of *Huangjiu* fermented with different brewing rice by using quantitative description sensory analysis and dynamic sensory evaluation techniques (TDS and TCATA) combined with electronic tongue; (2) to reveal the molecular difference of flavor compounds among the samples by using GC × GC-qMS and high performance liquid chromatography (HPLC), respectively; and (3) to further clarify the difference of aroma characteristics by multivariate statistical analysis, and to analyze the influence of brewing rice on the flavor characteristics by analyzing the correlation between the aroma-active compounds and sensory attributes among the samples. This study would improve the understanding of the influence of the brewing rice on the flavor compounds and provide a scientific basis for the selection of rice varieties to brew high-quality *Huangjiu*.

## 2. Materials and methods

### 2.1. Samples and chemicals

*Huangjiu* samples in Shaoxing region (S1, S2) fermented by glutinous rice and japonica rice, respectively, were provided by Zhejiang Pagoda Brand Shaoxing Rice Wine Co., Ltd., Shaoxing City, Zhejiang Province, China. The samples were brewed using the same Jianhu water, wheat *Qu* (a saccharification starter) and *Jiuyao* (a fermentation starter) with the same ratio. And the same brewing technique including soaking and steaming rice, fermentation (primary fermentation at 28°C for 3–5 days, then secondary fermentation at medium-low temperatures for about 90 days), filtration, pressing, clarification and storage was applied. All of the samples were stored at 4°C and they were analyzed within 1 month after being transferred to the laboratory.

The regents, aspartate, leucine, lysine, proline, histidine, arginine, 2-octanol (internal standard) and *n*-alkane standards (C_5_–C_30_) of chromatographic grade and purity ≥98.0% were obtained from Sigma Aldrich (Shanghai, China).

### 2.2. Sensory evaluation

The training and sensory analyzes were performed in a professional sensory laboratory at 20°C following ISO 8586-1:2012. Ten sensory panelists (5 males and 5 females, 23–27 years old) were selected from 40 candidates (20 males and 20 females, 23–30 years old) from the School of Perfume and Aroma Technology, Shanghai Institute of Technology (Shanghai, China). The taste sensory evaluation training was conducted for using the taste reference solution and Shaoxing *Huangjiu* samples for 4 weeks (1 h each time and 3 times a week). The panelists were specially trained to use taste description attributes and interval scale of 10 points for evaluation. During the taste sensory evaluation, *Huangjiu* sample (20 mL) was placed in a covered and odorless glass cup vial marked with a random three-digit code. The taste attributes included acidity (stated as the taste of citric acid aqueous solution), sweet (sucrose aqueous solution), bitter (quinine sulfate aqueous solution), astringent (alum aqueous solution), and umami (sodium glutamate aqueous solution). The intensities of the taste attributes were quantitatively determined on the interval scale of 10 points, where 0 indicated none and 9 indicated very intense. The methods and procedures of dynamic sensory evaluation including TCATA and TDS analysis were performed as described in our previous research (18).

Quantitative descriptive analysis (QDA) was similar to above taste sensory training, its methods and procedures were performed as described by our previous research (19). According to preliminary experiment discussion by the panelists, and referenced to a relevant literature (20), eight aroma attributes were selected, including sour (the aroma of acetic acid), sauce (4-ethylphenol), ester (ethyl acetate), sweet (vanillin), alcoholic (3-methylbutanol), caramel (caramel), fruit (ethyl isovalerate) and wheat (wheat *qu* aroma extract) aroma attributes. Prior to the formal sensory evaluation, the panelists were asked to smell each aroma attributes of standard reference sample and evaluated repeatedly according to the interval scale of 10 points. Additionally, for each one of the sensory evaluation indicated below, the sensory evaluation glasses used were ISO standard black glasses to eliminate the influence of sample color on the panelists. All sensory tests were conducted in triplicate, and a 10-min interval was maintained between each evaluation step.

### 2.3. Electronic tongue analysis

The TS-5000Z electronic tongue system (Insent Inc., Atsugi-Shi, Japan) including CAO, GL1, COO, AE1, AAE and Aftertaste-B sensors was used to collect the information of sour, sweet, bitter, astringent, fresh and post-bitter of the samples at 25°C. With tartric acid/potassium chloride solution as the reference solution, the sampling time was set to 120 s with the frequency was1 time/s, and each sample was were measured in triplicate.

### 2.4. Free amino acid analysis

Qualitative and quantitative analysis of 17 free amino acids in two kinds of *Huangjiu* samples was carried out by referring to QB/T 4356–2012 (National Standards of China). The C18 column (250 mm × 4.6 mm, 5 μm) was purchased from Agilent Technologies. The column temperature was maintained at 40°*C. mobile* phase A was 20 mmol/L sodium acetate buffer containing 0.05% v/v triethylamine. Mobile phase B was 80% acetonitrile and 20% water. The gradient elution procedure was as follows: 8–100% B from 0 to 33 min, 100% B from 33 to 36 min, 100–8% B from 36 to 38 min, and 8% B from 38 to 45 min. The injection volume was set to 10 μL, and the flow rate and detection wavelength were set to 1.0 mL/min and 254 nm, respectively. The amino acids in the samples were identified by comparing their retention times with those of the amino acid standards, and the concentration of each amino acid was analyzed according to the standard working curve of each amino acid standard solution.

### 2.5. Analysis of volatile compounds by solvent assisted flavor evaporation extraction combined with GC× GC-qMS

#### 2.5.1. Conditions of solvent assisted flavor evaporation

SAFE analysis was performed as described in our previous research (17), *Huangjiu* sample (60 mL), 200 μL internal standards (2-octanol, 315 μg/mL) and dichloromethane (60 mL) were placed in 250 mL conical flask, and extracted for 60 min at 250 r/min (20°C) in a shaker (ME104E, Mettler Toledo Instruments Co., Ltd., Shanghai, China). The extract was collected into a centrifuge tube (50 mL), and centrifuged at 4°C for 5 min (8,000 r/min) to collect the organic phase. The procedures of extraction were duplicated three times. The collected extract (about 180 mL) was combined, dried by nhydrous sodium sulfate and separated by SAFE apparatus (Glasbläserei Bahr, Manching, Germany). Liquid nitrogen was added to the cold trap and the turbine pump was turned on. When the required pressure (approximately 3 × 10^−3^ Pa) was reached, the sample was slowly and evenly controlled to drop into the distillation bottle. After extraction, the extract was dried and collected by rotary evaporation. Finally, the SAFE distillate was blown to 1 mL with nitrogen and stored at −20°C.

#### 2.5.2. Gas chromatography-quadrupole mass spectrometry analysis

The parameters and procedures for GC × GC-qMS (Shimadzu Co., Kyoto, Japan) analysis was described in our previous study (20, 21). An Agilent (Santa Clara, CA) HP-Innowax (60 m × 0.25 mm × 0.25 μm) and a Restek (Philadelphia, United States) BPX-1 (2 m × 0.1 mm × 0.1 μm) were used as column 1 and column 2, respectively. The column flow rate was 0.95 mL/min and split ratio was 20:1. The conditions were as follows: the oven temperature was held at 40°C and held for 5 min, then increased at 3°C/min to 150°C, finally increased at 4°C/min to 230°C. The temperature of column 2 was set to 5°C higher than that of column 1. And the modulation period was set to 8 s. The electron ionization energy was set to 70 eV, the ion source temperature was set at 200°C with high scanning frequency (20,000 Hz), and the total ion currents were recorded from m/z of 20 to 350. The analyzes were conducted in triplicate.

The volatile compounds were comprehensively characterized by GC-Image software (GC-Image LLC, Lincoln, NE), retention index and NIST 2014 (NIST, Gaithersburg-MD, United States) database and peak library matching factor. And the retention index was calculated based on C_5_-C_30_ alkane standards (Sigma-Aldrich, St. Louis, MO, United States).

### 2.6. Odor activity values calculation

The contribution of a certain compound to the aroma characteristics of the samples was evaluated by its OAV (ratio of the mass concentration and the odor threshold value) (17). The odor threshold values were taken from available information in the compilation (22) and based on water or ethanol solution matrix. The characteristic aroma compounds in the samples were screened according to OAV > 1.

### 2.7. Statistical analysis

Sensory data were analyzed by Duncan’s multiple range tests using SPSS Statistics 21 (SPSS Inc., Chicago, United States). Heat map was generated by using the pheatmap package of the R program. The multivariate analyzes were performed by SIMCA-PTM14.1 (UMetrics AB, Umea, Sweden) software.

## 3. Results and discussion

### 3.1. Taste characteristics analysis of *Huangjiu* fermented with different brewing rice

**Figure 1:**
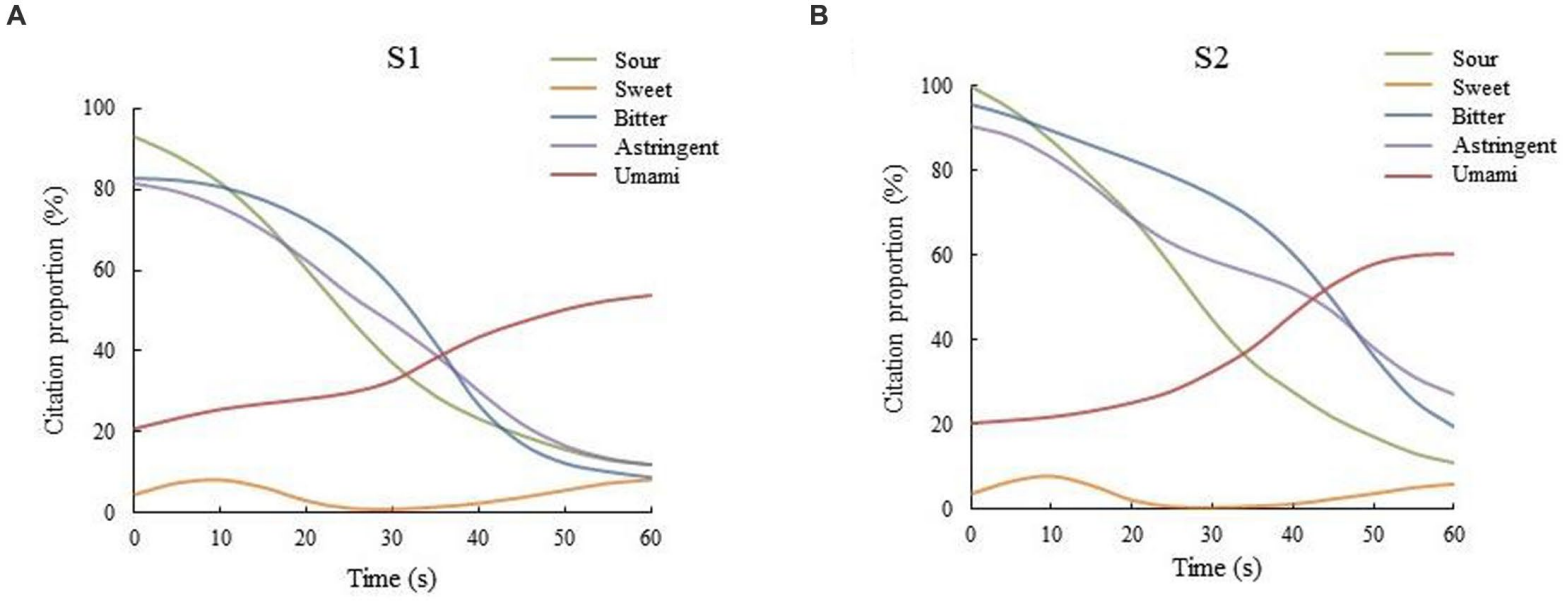
TCATA curves of the taste of the two types of *Huangjiu* samples fermented with different rice varieties (**A**,**B** represent the *Huangjiu* fermented with glutinous rice and japonica rice, respectively).

In the process of drinking, the taste attributes were not static, and the dynamic changes of tastes will also affect the judgment on the taste quality of *Huangjiu*. Therefore, the taste properties of *Huangjiu* samples fermented with different brewing rice were analyzed by dynamic sensory evaluation methods including TCATA and TDS. By collecting the citation proportion of each taste attribute of the samples within 60s, the TCATA test can intuitively reflect the dynamic changes of taste attributes of the samples with time during drinking. As shown in Figure 1, the variation trends of the taste attributes of *Huangjiu* samples fermented with different brewing rice were basically similar. Among them, the taste attributes of *Huangjiu* samples fermented with glutinous rice and japonica rice that were noted more than 50% of the time were mainly sour, bitter and astringent at the early stage, and sweet and umami in the late stage. The citation proportion of sour, bitter and astringent tastes in two kinds of *Huangjiu* samples gradually decreased after drinking, while the umami taste was the opposite, which may be related to the fact that the sauce flavor in *Huangjiu* was transformed into post-nose taste after being drunk in the mouth, thus gradually enhancing the perception of umami taste of the panelists (23). Complex oral processing can also enhance the umami taste in the middle and late stages of digestion (24). On the whole, the citation proportion of taste attributes of the *Huangjiu* fermented with japonica rice was slightly higher than that of the *Huangjiu* fermented with glutinous rice, and the taste characteristic was relatively richer. However, the taste of the former was more sour and bitter, and the citation proportion of bitter at the end of the 60s test was 19.5%, which was also significantly higher than that of S1 sample (8.9%).

**Figure 2:**
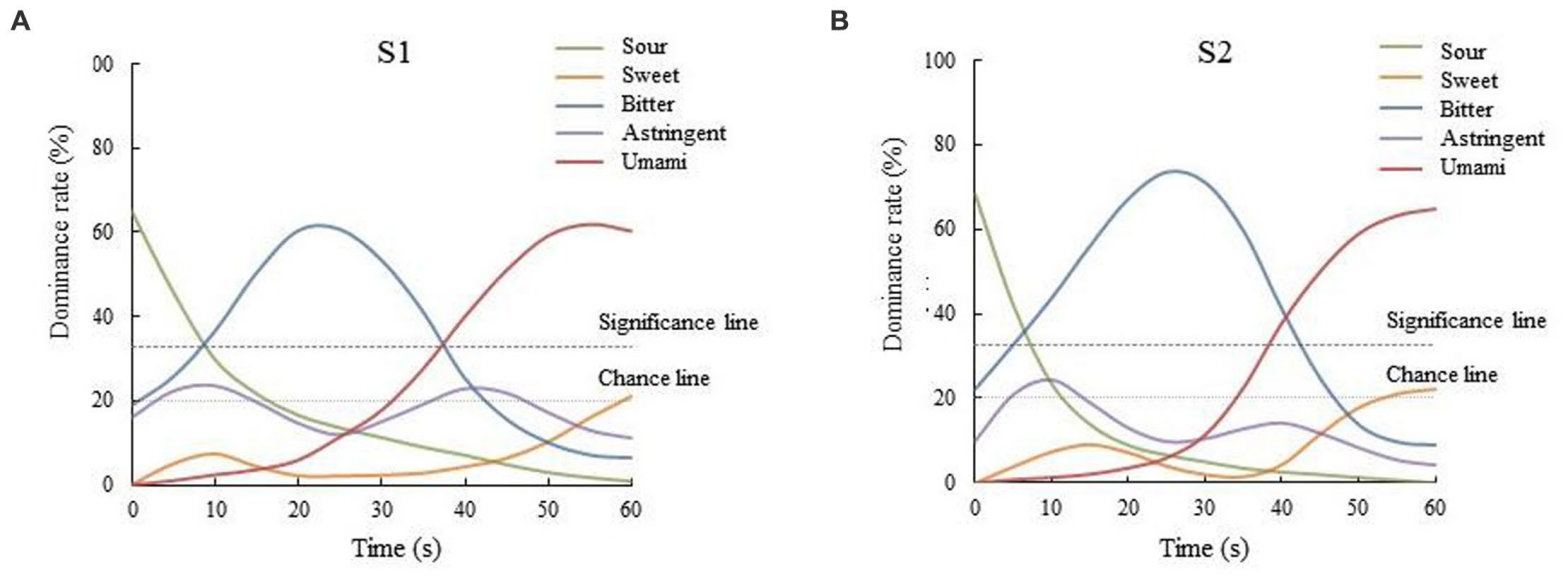
TDS curves of the taste of the two types of *Huangjiu* samples fermented with different rice varieties (A and B represent the *Huangjiu* fermented with glutinous rice and japonica rice, respectively).

Compared with TCATA, a sensory analysis method that is close to consumer testing (25), the TDS method can better reflect the influence of some dominant taste attributes on sensory attributes during drinking. The dominance rates of taste attributes of two types of *Huangjiu* samples within 60s after drinking were collected to clarify the changes of dominant taste attributes. The TDS analysis results among the *Huangjiu* samples are shown in Figure 2. The taste attributes of the two types of *Huangjiu* samples had similar dynamic trends that the order of sour, bitter and umami was maintained. At the beginning of the test (0 s), the sour taste dominated in the two types of *Huangjiu* samples. In the first 20s, with the decrease of the dominant rate of sour taste over time, the dominance of bitter taste gradually increased, which may be related to the symmetrical inhibition of sour and bitter. After 20s of the TDS test, the dominant rates of sour and bitter tastes among the samples gradually decreased, while umami taste broke through the chance level and significance level, and then occupied the dominant taste attribute. The curve above the line of chance level indicated that taste attributes can be accidentally perceived, and the significance level revealed that the cut-off above, the probability of taste attributes being selected was significantly greater than chance (26). The above feature was not only consistent with the taste characteristics of umami taste in the late stage, but also related to the inhibition of umami taste on sour and bitter taste (27). In general, although the variation trends of the taste attributes during drinking among the samples were basically similar, the bitter taste maintained shorter time and lower dominance rate in the overall drinking process of *Huangjiu* fermented with glutinous rice, indicating that the bitterness of *Huangjiu* fermented with glutinous rice was lighter and easier to dissipate than that of *Huangjiu* fermented with japonica rice, and the overall palatability was higher than that of the latter.

To further analyze the taste of *Huangjiu* samples and test the results of TCATA and TDS, the taste of two types of *Huangjiu* fermented by different brewing rice was evaluated by intelligent sensory electronic tongue technology. The taste evaluation of *Huangjiu* samples was shown in Table 1. The response values of astringency and post-bitterness taste of S1 sample were weaker than those of S2 sample (*p* < 0.05), but there were no difference in other taste attributes. Additionally, the response value of umami taste was slightly better than the latter, which was consistent with the above dynamic sensory test results. The combination of artificial sensory evaluation and electronic tongue was conducive to fully characterize the taste evaluation of *Huangjiu*.

**Table 1:** Taste evaluation of *Huangjiu* fermented with different rice varieties by electronic tongue.

	Sour	Sweet	Bitter	Astringent	Umami	After-bitterness
S1	6.47 ± 0.634a	−5.23 ± 0.287a	5.52 ± 0.376a	1.22 ± 0.0143b	9.61 ± 0.528a	0.609 ± 0.0275b
S2	6.64 ± 0.620a	−5.48 ± 0.241a	5.74 ± 0.289a	1.35 ± 0.0256a	9.39 ± 0.633a	0.632 ± 0.0240a

### 3.2. Free amino acids analysis of *Huangjiu* fermented with different brewing rice

*Huangjiu* contained a large amount of amino acids, which endowed *Huangjiu* with a rich taste level such as delicious, soft and mellow. The concentrations of free amino acids determined by high performance liquid chromatography (HPLC) in two kinds of *Huangjiu* samples are shown in Table 2. The total content of free amino acids in *Huangjiu* fermented with japonica rice (S2) was slightly higher than that in *Huangjiu* fermented with glutinous rice (S1), which was related to the higher protein content in japonica rice. The concentrations of free amino acids imparting sweet, bitter, umami, astringent and sour tastes were significantly different among the samples. The contents of aspartic acid and glutamic acid in S1 sample were higher, which played an important contribution to the umami and mellow taste. In addition, bitter amino acids were found to account for more than 40% of the total amino acids in the *Huangjiu* samples (40.2–43.9%), which was consistent with that of Liang et al. (28). Among them, the concentrations of histidine and leucine with bitter taste in S2 sample were higher than those of S1 sample, while isoleucine, phenylalanine and arginine were lower than the latter. Arginine imparted little bitter tastes, and bitterness was enhanced at high concentrations.

**Table 2:** Analysis of free amino acids of *Huangjiu* fermented with different rice varieties (mg/kg).

Free amino acid	S1	S2
Sweet		
Serine (Ser)	98.6 ± 1.63a	98.8 ± 1.02a
Glycine (Gly)	202 ± 2.34b	212 ± 1.66a
Threonine (Thr)	124 ± 2.04a	124 ± 1.35a
Alanine (Ala)	430 ± 4.89b	461 ± 2.04a
Proline (Pro)	499 ± 5.93b	614 ± 6.19a
Methionine (Met)	53.3 ± 3.23a	59.5 ± 3.10a
Bitter		
Histidine (His)	91.6 ± 1.15b	95.9 ± 1.09a
Arginine (Arg)	600 ± 5.51a	526 ± 4.08b
Valine (Val)	235 ± 4.49a	225 ± 1.22b
Isoleucine (Ile)	143 ± 1.87a	140 ± 2.66a
Leucine (Leu)	387 ± 4.89a	390 ± 2.55a
Phenylalanine (Phe)	235 ± 4.08a	194 ± 2.51b
Lysine (Lys)	180 ± 2.33a	167 ± 1.86b
Umami		
Aspartic acid (Asp)	277 ± 1.86a	273 ± 2.67b
Glutamate (Glu)	422 ± 3.72a	420 ± 5.31a
Astringent		
Tyrosine (Tyr)	280 ± 1.98a	313 ± 3.31b
Sour		
Cysteine (Cys)	7.93 ± 0.883a	10.3 ± 0.577b

Values with different letters (a–b) in a row are significantly different using Duncan’s multiple comparison tests (*p* < 0.05).

The umami and sweet taste of tea soup had a positive correlation, while the umami and bitter taste negatively correlated (29). The same taste interaction may also exist in *Huangjiu*, where the umami taste provided by glutamic acid and aspartic acid may enhance the perception of sweet taste and weaken the perception of bitter taste. Therefore, the perception of sweet taste in S1 sample was stronger than that of S2 sample, while the bitterness perception was weaker. The contents of sour and astringent amino acids in *Huangjiu* samples fermented with glutinous rice were significantly lower (*p* < 0.05), which was consistent with the results of electronic tongue and artificial sensory tests on the whole.

### 3.3. Quantitative descriptive sensory analysis of *Huangjiu* fermented with different brewing rice

**Figure 3:**
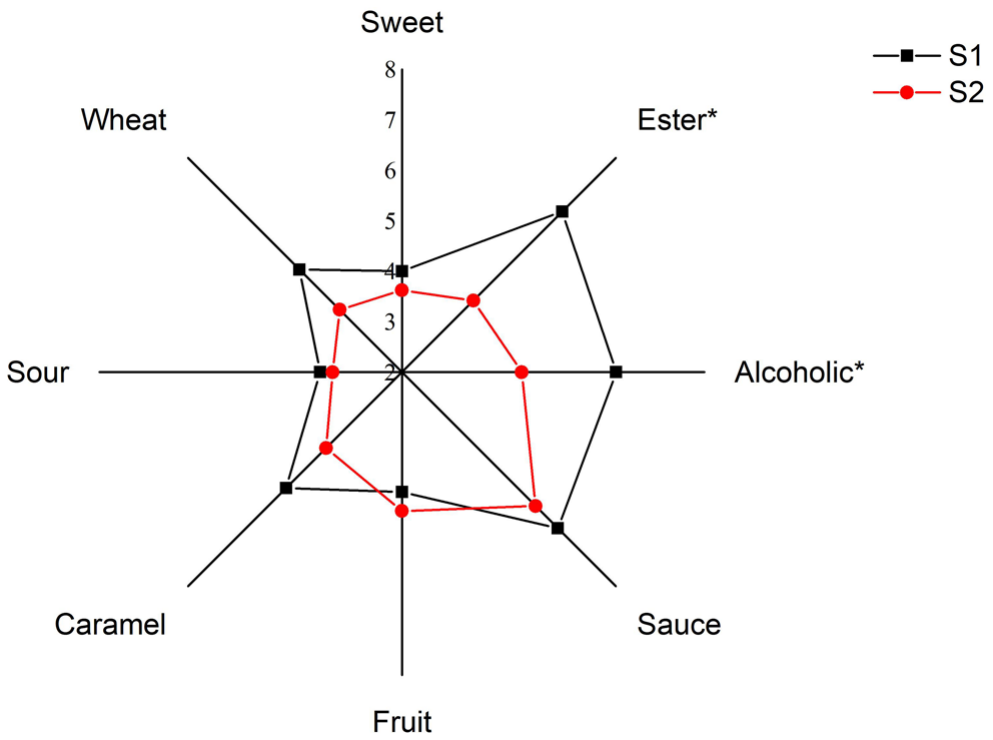
Quantitative descriptive sensory radar chart of *Huangjiu* fermented with different different rice varieties (*represents *p* < 0.05, S1 and S2 correspond to the *Huangjiu* samples fermented by glutinous rice and japonica rice, respectively).

To determine the aroma difference among the samples fermented with different brewing rice, quantitative descriptive analysis was analyzed and the results were shown in Figure 3. Ester, alcoholic, sauce and fruit aroma were the representative aroma in the two kinds of *Huangjiu*. Statistical analysis showed that the attributes of ester and alcoholic aroma in S1 sample were significantly higher (*p* < 0.05). Additionally, the scores of sweet, sauce, caramel and sour aroma were abundant, while there was no significant difference between these aroma attributes, which was consistent with the research of Chen et al. (19). The results of sensory evaluation indicated that *Huangjiu* fermented with glutinous rice was more prominent in ester and alcoholic aroma, and had better aroma quality.

### 3.4. Aroma components analysis of *Huangjiu* fermented with different brewing rice by GC × GC-qMS

**Figure 4:**
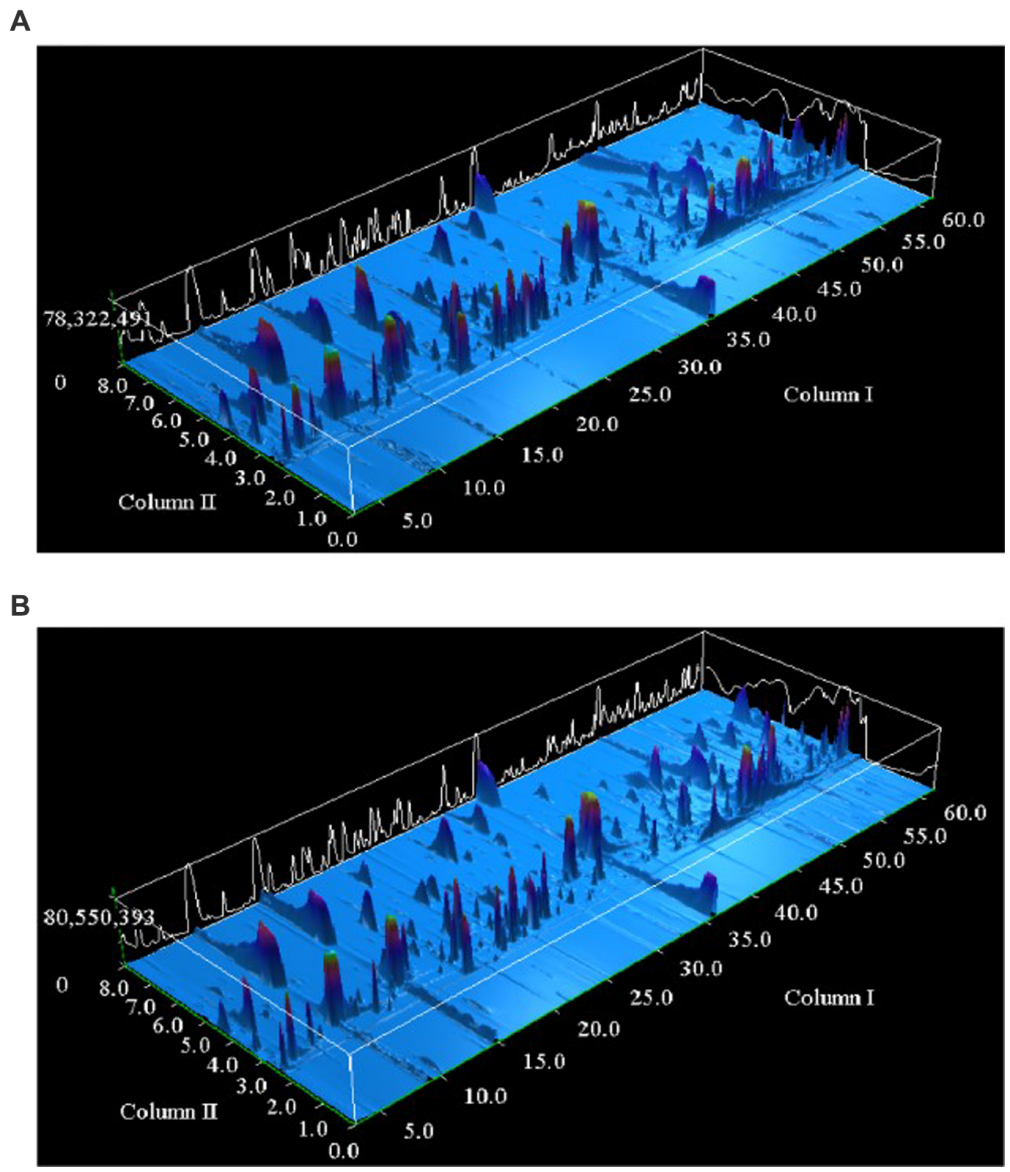
Three dimensional chromatograms of volatile components of Huangjiu fermented with **(A)** glutinous rice and **(B)** japonica rice were determined by GC × GC/qMS.

The volatile characteristics of *Huangjiu* fermented with different brewing rice were systematically analyzed by GC × GC-qMS. The three-dimensional chromatograms of the volatile distribution of two kinds of *Huangjiu* samples are shown in Figure 4. DB-INNOWAX column was used to successfully separate the volatile compounds in the first dimension, and the BPX-5 column was also successfully separated in the second dimension. The aroma components of the *Huangjiu* samples were analyzed and their results were listed in Supplementary Table S1. A total of 111 volatile compounds were identified, of which 101 and 99 were identified in S1 sample and S2 sample, respectively, which consisted of 32 esters, 20 alcohols, 12 aldehydes, 19 acids, 9 ketones, 5 phenols, 1 ether, 6 amines, 1 epoxide and 6 other compounds. Although the composition of volatile compounds in the two samples was basically similar, there were obvious differences in the concentrations of specific compounds.

The content of esters in the *Huangjiu* samples fermented with different brewing rice was abundant, and most of which were ethyl esters. Ethyl ester was mainly formed by esterification of fatty acids and ethanol (30) and endowed *Huangjiu* with fruity, sweet and floral aroma. Among the esters detected, monoethyl succinate, ethyl 3-hydroxybutyrate, diethyl succinate, ethyl butyrate, propyl nonolactone and ethyl caprylate were the major esters with the highest concentrations. Among them, the OAVs of ethyl butyrate (OAV: 38–59), ethyl caprylate (OAV: 30–38) and propyl nonolactone (OAV: 4–7) were all greater than 1. These three compounds had apple, banana and peach aroma, respectively, indicating they were important compounds that affected the aroma characteristics of *Huangjiu*. In addition, isoamyl acetate (OAV = 5) and sugar lactone (OAV = 6) with low concentrations were only indentified in S1 sample. The compound isoamyl acetate contributed to sweet and fruity aroma and was an important precursor for the formation of many aroma compounds. The compound sugar lactone was a key compound for the aroma characteristics of *Huangjiu*, which endowed *Huangjiu* with caramel aroma at low concentrations while curry aroma at high concentrations (31). This result was consistent with the scores of sweet and caramel aroma in descriptive sensory analysis. However, the compound 2-methylbutyl acetate (OAV = 14) with low concentration was only detected in S2 sample and was a characteristic aroma compound endowing *Huangjiu* with fruity aroma.

Alcohols were the important source of aroma in *Huangjiu* and were precursors of esters, which endowed *Huangjiu* with mellow and sweet. The concentrations of total alcohols in S1 sample (93.35–101.92 μg/g) were significantly higher than those in S2 sample (84.92–94.97 μg/g). This trend may be related to the higher content of amylopectin in glutinous rice, and the easy gelatinization of amylopectin, which promoted the conversion of sugars to alcohols during *Huangjiu* fermentation and thus improving the alcohol yield. Among the alcohols detected, 3-methylbutanol had the highest content, followed by phenylethanol, isobutanol, 2,3-butanediol, 3-methylthiopropanol and 1-butanol. The sum of these six alcohols accounted for 97.2–97.3% of the total alcohols among the samples. Among them, the OAVs of phenylethyl alcohol (OAV: 3–4) and 3-methylthiopropanol (1–3) were both greater than 1. The compound phenylethyl alcohol with rose and honey aromas can be synthesized by pentose phosphate or glycolytic pathway (32), and under anaerobic conditions, valine and phenylalanine can be converted into isobutanol and phenylethanol, respectively (33). The compound 3-methylthiopropanol with sweet and potato aromas may be derived from the degradation of sulfur-containing amino acids.

Aldehydes and ketones were conducive to enhancing the aroma and soft taste of *Huangjiu*. Most aldehydes were produced by the oxidation of higher alcohols or Maillard reaction during *Huangjiu* fermentation. The content of aldehydes in S1 sample was higher than that in S2 sample. The main aldehydes with high concentrations in the samples were n-hexanal, furfural and 5-methyl furfural. The concentrations of furfural and syringaldehyde with sweet taste in S1 sample were higher than that in S2 sample, while nonaldehyde, benzaldehyde and phenylacetaldehyde in S2 sample were significantly higher than that in S1 sample. Among them, the compound phenylacetaldehyde (OAV = 292) with sweet and floral aroma was only detected in S2 sample, and was an important odorant in *Huangjiu* (34). In S1 sample, the compound 3-hydroxy-2-butanone with sweet and creamy aroma had the highest concentration, which was the key compound for the synthesis of 2,3-butanedione and 2,3-butanediol. Additionally, 4-ethylphenol, 4-vinyl-2-methoxyphenol and guaiacol had high relative concentrations. Among them, the compound 4-ethylphenol endowed a smoky aroma (35) and was only detected in S1 sample. And the concentration of 4-vinyl-2-methoxyphenol with clove aroma in S1 sample was much higher than that in S2 sample. The abundance of acids increased the body of *Huangjiu* and facilitated the formation of aromatic esters. Appropriate amount of acids can also increase the sweet taste and weaken the bitterness of wine, which may be the reason why *Huangjiu* fermented with glutinous rice was less bitter and astringent than *Huangjiu* fermented with japonica rice. A total of 19 acid compounds were detected in the samples. The contents of acids in S1 sample (11.31–11.34 μg/g) were significantly higher than those in S2 sample (8.31–7.49 μg/g). This may be due to the low protein content and dispersed structure in glutinous rice (36). Among them, the concentration of butyric acid was the highest, followed by acetic acid, palmitic acid and hexanoic acid. The production of these organic acids were closely related to the raw materials in *Huangjiu* brewing, including the process of alcohol fermentation or aging (37).

In conclusion, the total contents of alcohols, aldehydes, acids and characteristic flavor compounds in *Huangjiu* sample fermented with glutinous rice were higher than those of *Huangjiu* sample fermented with japonica rice. The compound ethyl butyrate, isoamyl acetate, 3–methylthiopropionaldehyde and ethyl caprylate were the characteristic flavor compounds of S1 sample, while nonanal, phenyl acetaldehyde and vanillin contributed greatly to the flavor of S2 sample.

### 3.5. Differences of aroma characteristics among the samples with different brewing rice

**Figure 5:**
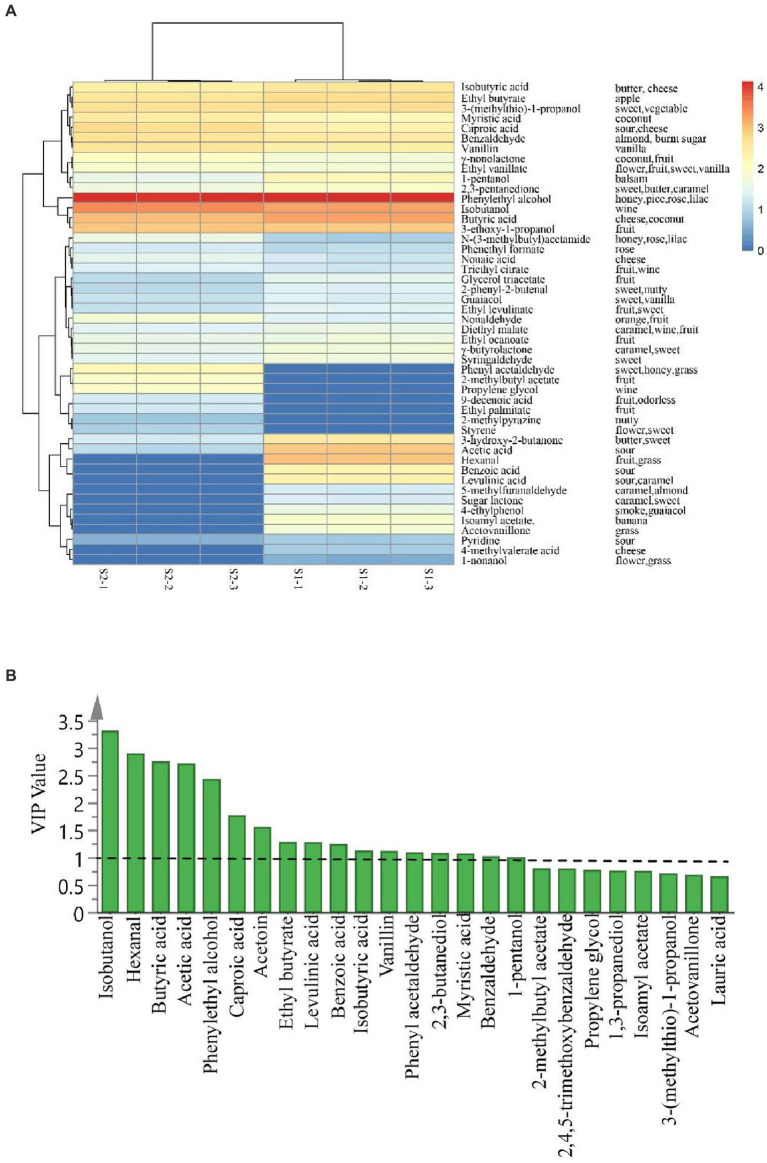
Heat map and HCA clustering results of 48 aroma compounds with a significant difference (p < 0.05) and aroma descriptors in *Huangjiu* samples **(A)** (S1 and S2 correspond to the *Huangjiu* samples fermented by glutinous rice and japonica rice, respectively); The VIP plot of the aroma compounds based on the OPLS-DA regression model **(B)**.

To fully explore the data of aroma compounds obtained by GC × GC-qMS, one-way ANOVA was performed on 111 identified volatile compounds. The results showed that there were significant differences in 64 compounds among the samples. Among them, 48 compounds had clear aroma descriptions, including 15 esters, 6 alcohols, 7 aldehydes, 10 acids, 2 phenols, 1 ether, 2 ketones, 4 aromatics and 1 amine compound. The correlation between the samples and the 48 important aroma compounds (*p* < 0.05) with aroma description was visualized by heatmap, and the results were shown in Figure 5A.

The VIP values were usually used to assess the contributions of X-variables to the model, and variables with VIP > 1 were considered important variables (Figure 5B). The VIP values of 17 volatile compounds were greater than 1, including 7 acids (butyric acid, acetic acid, hexanoic acid, levulinic acid, benzoic acid, isobutyric acid and myristic acid), 4 alcohols (isobutanol, phenethyl alcohol, 2,3-butanediol and 1-pentanol), 3 aldehydes (hexanal, phenylacetaldehyde and benzaldehyde), ethyl butyrate, acetoin and vanillin. These volatile compounds could be conducive to distinguishing the *Huangjiu* samples fermented with different rice varieties.

### 3.6. Correlation analysis between the aroma compounds and sensory attributes in *Huangjiu* samples

**Figure 6:**
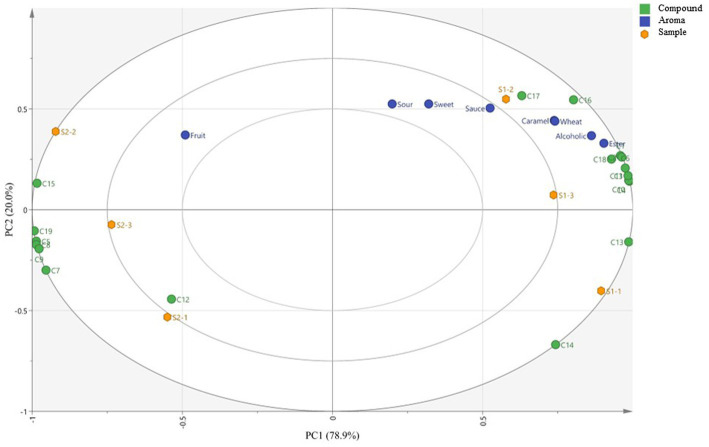
Correlation of aromas with aroma-active compounds. Yellow dots represent two *Huangjiu* samples. Blue dots represent 7 aroma attributes. Green dots represent the active-aroma compounds with OAV >1 shown in Supplementary Table S1 (C1: ethyl butyrate, C2: 3-penten-2-one, C3: isoamyl acetate, C4: 1-pentanol, C5: nonanal, C6: butyric acid, C7: γ-nonanolactone, C8: Phenyl acetaldehyde, C9: vanillin, C10: hexanal, C11: sugar lactone, C12: 3-ethoxy-1-propanol, C13: 3-(methylthio)-1-propanol. C14: phenylethyl alcohol, C15: ethyl hexanoate, C16: 2-octanone, C17: ethyl caprylate, C18: 3-(methylthio)propionaldehyde, C19: 2-methylbutyl acetate).

To further determine the correlation between the aroma compounds and sensory attributes, and analyze their contributions to the aroma of the *Huangjiu* samples fermented with different rice varieties, PLS regression was performed and the results are shown in Figure 6. The values of cumulated R^2^X (0.907) and R^2^Y (0.902) corresponding to the relationships between the explanatory variables (X, aroma-active compounds) and dependent variables (Y, intensity of the aromas of the compounds) were close to 1. And the model quality (Q^2^ = 0.701) was appropriate as Q^2^ > 0.50, indicating that the correlation between the two variables can be well represented by PLS analysis.

S1 sample was correlated with ‘ester’, ‘alcoholic’, ‘wheat’, ‘caramel’, ‘sauce’, ‘sweet’ and ‘sour’ aromas, while S2 sample was correlated with ‘fruit’ aroma. And the volatile compounds ethyl octanoate and 2-octanone were strongly associated with ‘sauce’, ‘caramel’, ‘wheat’ and ‘alcoholic’ aromas. The ‘ester’ and ‘alcoholic’ aromas were strongly associated with most compounds, including ethyl butyrate, 3-penten-2-one, 3-(methylthio)propanal, isoamyl acetate, and so on. Collcetively, most aroma-active compounds were correlated with ‘ester’ aroma in *Huangjiu* sample fermented with glutinous rice, which was consistent with the results of QDA analysis.

## 4. Conclusion

The effect of different rice varieties on the taste and aroma characteristics of *Huangjiu* was investigated using dynamic sensory evaluation, GC × GC-qMS and multivariate statistical analysis. The results revealed that the flavor characteristics and profiles of two kinds of *Huangjiu* had remarkable differences. Compared with *Huangjiu* fermented with japonica rice, the tastes of bitter and astringency in *Huangjiu* fermented with glutinous rice were weaker, and the ester and alcoholic aromas were more prominent. Ethyl butyrate, isoamyl acetate, 3–methylthiopropionaldehyde and ethyl caprylate contributed greatly to the flavor of *Huangjiu* sample fermented with glutinous rice, while nonanal, phenyl acetaldehyde and vanillin were the characteristic flavor compounds in *Huangjiu* sample fermented with japonica rice. Furthermore, the total contents of alcohols, acids and aldehydes were higher in the *Huangjiu* sample fermented with glutinous rice, and the important aroma compounds (especially key esters) were more abundant. Correlation analysis further proved that most aroma-active compounds significantly correlated with ester and alcoholic aroma in the *Huangjiu* sample fermented with glutinous rice. The comprehensive analysis of taste and aroma characteristics showed that the *Huangjiu* fermented by glutinous rice had higher flavor quality. Our results would provide a certain guiding effect on the quality control and taste improvement of *Huangjiu*. However, the exact factors that cause the differences in aroma and taste of the two kinds of *Huangjiu* are still unclear, and further research from the microbial perspective is needed.

## Data availability statement

The original contributions presented in the study are included in the article/Supplementary material, further inquiries can be directed to the corresponding author.

## Author contributions

HY: conceptualization, methodology, formal analysis, resources, writing–original draft, and writing–review and editing. QL: methodology, formal analysis, investigation, and writing–original draft. WG: methodology and formal analysis. LA: resources and supervision. CC: resources, supervision, and project administration. HT: writing–review and editing and project administration. All authors contributed to the article and approved the submitted version.

## Funding

The research was supported by the National Natural Science Foundation of China (No. 32172336) and the Capacity Project of Local Colleges and Universities of the Science and Technology Commission of Shanghai, China (No. 21010504100).

## Conflict of interest

The authors declare that the research was conducted in the absence of any commercial or financial relationships that could be construed as a potential conflict of interest.

## Publisher’s note

All claims expressed in this article are solely those of the authors and do not necessarily represent those of their affiliated organizations, or those of the publisher, the editors and the reviewers. Any product that may be evaluated in this article, or claim that may be made by its manufacturer, is not guaranteed or endorsed by the publisher.
