# Supplementary material for: Unraveling the difference in flavor characteristics of Huangjiu fermented with different rice varieties using dynamic sensory evaluation and comprehensive two-dimensional gas chromatography–quadrupole mass spectrometry
Source: Front Nutr. 2023 Jun 22;10:1160954. doi: 10.3389/fnut.2023.1160954 (PMC10324613; doi:10.3389/fnut.2023.1160954)
Supplement: Supplementary file 1 [file Table_1.DOCX]

**Supplementary Table 1**

The concentrations (μg/g) and standard deviation of volatile compounds detected by GC×GC-qMS in *Huangjiu* fermented with different brewing rice.

| No. | Compound | LRI  calculated^a^ | LRI  Reported^b^ | Library match factor | 1tR(min)^c^ | 2tR(s)^d^ | Concentration (μg g^-1^) | | OAV | |
| --- | --- | --- | --- | --- | --- | --- | --- | --- | --- | --- |
|  |  |  |  |  |  |  | S1 | S2 | S1 | S2 |
| 1 | Ethyl butyrate | 1019 | 1030 | 858 | 3.53 | 4.34 | 1.176±0.072a^e^ | 0.767±0.012b | 59 | 38 |
| 2 | 1-propanol | 1030 | 1037 | 878 | 3.80 | 3.38 | 0.818±0.043a | 0.881±0.028a | <1 | <1 |
| 3 | 2-methyl-m-dioxane | 1025 | 1044 | 801 | 3.67 | 3.50 | 0.079±0.003a | 0.157±0.003b | -^f^ | - |
| 4 | 2,3-pentanedione | 1030 | 1056 | 729 | 3.80 | 2.94 | 0.037±0.003a | 0.061±0.005b | <1 | <1 |
| 5 | Isobutanol | 1100 | 1099 | 821 | 5.40 | 4.04 | 4.343±0.363a | 7.002±0.503b | <1 | <1 |
| 6 | 3-penten-2-one | 1100 | 1121 | 828 | 5.40 | 6.18 | 0.008±0.001 | - | 5 | - |
| 7 | Isoamyl acetate | 1104 | 1129 | 772 | 5.53 | 0.72 | 0.141±0.004 | - | 5 | - |
| 8 | 1-butanol | 1137 | 1135 | 867 | 6.73 | 4.18 | 1.006±0.053a | 0.952±0.040a | <1 | <1 |
| 9 | 3-methylbutanol | 1211 | 1212 | 833 | 9.40 | 6.72 | 47.817±3.923a | 40.264±4.024a | 1 | 1 |
| 10 | 1-pentanol | 1253 | 1254 | 872 | 10.87 | 3.12 | 0.303±0.018a | 0.056±0.001b | 2 | <1 |
| 11 | Acetoin | 1276 | 1265 | 809 | 11.67 | 4.12 | 0.616±0.039a | 0.027±0.002b | <1 | <1 |
| 12 | Hydroxyacetone | 1295 | 1301 | 813 | 12.33 | 5.52 | 0.023±0.004a | 0.026±0.001a | <1 | <1 |
| 13 | 1-hexanol | 1353 | 1356 | 865 | 15.27 | 3.68 | 0.451±0.037a | 0.445±0.022a | <1 | <1 |
| 14 | 3-ethoxy-1-propanol | 1361 | 1359 | 790 | 15.67 | 5.90 | 1.829±0.040a | 1.908±0.115a | 18 | 19 |
| 15 | Nonanal | 1379 | 1388 | 891 | 16.60 | 4.42 | 0.035±0.003a | 0.108±0.007b | 2 | 7 |
| 16 | Ethyl glycolate | 1403 | 1436 | 841 | 17.80 | 2.82 | 0.023±0.003a | 0.025±0.001a | - | - |
| 17 | 3-(methylthio)propionaldehyde | 1442 | 1418 | 794 | 19.00 | 3.46 | 0.048±0.009a | 0.023±0.003a | 96 | 47 |
| 18 | Ethyl caprylate | 1442 | 1442 | 872 | 19.00 | 6.20 | 0.077±0.020a | 0.060±0.002a | 38 | 30 |
| 19 | Isobutyl lactate | 1451 | 1455 | 794 | 19.27 | 4.38 | 0.148±0.006a | 0.183±0.011a | <1 | <1 |
| 20 | Furfural | 1455 | 1445 | 854 | 19.40 | 3.00 | 1.015±0.070a | 0.886±0.027a | <1 | <1 |
| 21 | 2-ethylhexanol | 1494 | 1493 | 885 | 20.60 | 5.32 | 0.024±0.007a | 0.016±0.002a | <1 | <1 |
| 22 | Benzaldehyde | 1510 | 1518 | 896 | 21.27 | 3.92 | 0.623±0.005a | 0.882±0.065b | <1 | <1 |
| 23 | Ethyl 3-hydroxybutyrate | 1516 | 1521 | 785 | 21.53 | 3.68 | 2.591±0.253a | 3.138±0.195b | <1 | <1 |
| 24 | 2-methylthioethanol | 1521 | 1537 | 869 | 21.80 | 2.94 | 0.021±0.005a | 0.024±0.001a | <1 | <1 |
| 25 | Propionic acid | 1538 | 1531 | 807 | 22.60 | 2.50 | 0.211±0.023a | 0.151±0.014a | <1 | <1 |
| 26 | Ethyl2-hydroxy-4-methylvalerate | 1541 | 1515 | 811 | 22.73 | 5.36 | 0.468±0.030a | 0.482±0.009a | - | - |
| 27 | Isoamyl lactate | 1561 | 1558 | 818 | 23.67 | 5.38 | 0.396±0.029a | 0.501±0.038a | - | - |
| 28 | Isobutyric acid | 1561 | 1544 | 804 | 23.67 | 3.80 | 0.799±0.010a | 0.490±0.012b | <1 | <1 |
| 29 | 2,3-butanediol | 1541 | 1553 | 860 | 22.73 | 4.92 | 1.891±0.163a | 1.709±0.137a | <1 | <1 |
| 30 | Butyric acid | 1614 | 1639 | 686 | 25.93 | 2.82 | 4.297±0.192a | 2.445±0.133b | 2 | 1 |
| 31 | Ethyl methyl succinate | 1627 | 1631 | 853 | 26.33 | 5.16 | 0.014±0.001a | 0.015±0.001a | - | - |
| 32 | Ethyl benzoate | 1648 | 1681 | 830 | 27.00 | 6.68 | 0.012±0.001a | 0.015±0.001a | <1 | <1 |
| 33 | γ-hexalactone | 1678 | 1683 | 831 | 27.93 | 3.98 | 0.016±0.001a | 0.019±0.002a | <1 | <1 |
| 34 | Diethyl succinate | 1678 | 1687 | 849 | 27.93 | 6.56 | 2.246±0.177a | 2.718±0.210a | <1 | <1 |
| 35 | 3-(methylthio)-1-propanol | 1711 | 1736 | 828 | 29.00 | 3.36 | 1.115±0.038a | 0.983±0.046b | 2 | 2 |
| 36 | Valeric acid | 1741 | 1734 | 810 | 30.07 | 2.78 | 0.077±0.005a | 0.065±0.005a | <1 | <1 |
| 37 | 3,3-dimethylacrylic acid | 1796 | 1780 | 843 | 32.07 | 2.76 | 0.015±0.001a | 0.015±0.001a | <1 | <1 |
| 38 | 4-methylvaleric acid | 1803 | 1800 | 772 | 32.33 | 5.96 | 0.010±0.001 | - | <1 | <1 |
| 39 | Caproic acid | 1833 | 1831 | 841 | 33.67 | 5.22 | 0.415±0.024a | 1.179±0.129b | <1 | <1 |
| 40 | Guaiacol | 1839 | 1859 | 818 | 33.93 | 3.86 | 0.032±0.003a | 0.019±0.002b | 1 | 1 |
| 41 | Benzyl alcohol | 1853 | 1878 | 871 | 34.60 | 3.36 | 0.162±0.006a | 0.213±0.012b | <1 | <1 |
| 42 | N-isoamylacetamide | 1862 | 1866 | 847 | 35.00 | 4.04 | 0.011±0.002a | 0.068±0.004b | - | - |
| 43 | Phenylethyl alcohol | 1880 | 1872 | 869 | 35.80 | 8.10 | 38.870±2.714a | 36.503±2.678a | 4 | 4 |
| 44 | Enanthic acid | 1925 | 1950 | 849 | 37.40 | 3.38 | 0.011±0.001a | 0.015±0.002a | - | - |
| 45 | γ-nonanolactone | 1987 | 2008 | 840 | 39.13 | 6.94 | 0.107±0.010a | 0.180±0.005b | 4 | 7 |
| 46 | DL-pantolactone | 2001 | 2034 | 830 | 39.53 | 3.06 | 0.281±0.012a | 0.292±0.016a | - | - |
| 47 | Caprylic acid | 2040 | 2039 | 873 | 40.73 | 3.86 | 0.297±0.014a | 0.343±0.009a | <1 | <1 |
| 48 | Triacetin | 2066 | 2077 | 858 | 41.53 | 5.46 | 0.047±0.000a | 0.016±0.001b | - | - |
| 49 | Nonaic acid | 2153 | 2173 | 893 | 43.93 | 6.90 | 0.024±0.004a | 0.048±0.001b | <1 | <1 |
| 50 | 4-ethylphenol | 2159 | 2196 | 733 | 44.07 | 6.26 | 0.072±0.005 | - | <1 | - |
| 51 | Decanoic acid | 2256 | 2265 | 789 | 46.60 | 4.16 | 0.106±0.004a | 0.160±0.010b | <1 | <1 |
| 52 | Benzoic acid | 2408 | 2448 | 832 | 50.07 | 5.80 | 0.379±0.030 | - | <1 | - |
| 53 | Triethyl citrate | 2456 | 2461 | 821 | 51.13 | 5.84 | 0.025±0.001a | 0.035±0.002b | - | - |
| 54 | Lauric acid | 2474 | 2503 | 886 | 51.53 | 4.68 | 0.127±0.010a | 0.236±0.020b | <1 | <1 |
| 55 | 5-hydroxymethylfurfural | 2480 | 2487 | 854 | 51.67 | 2.84 | 0.711±0.018a | 0.769±0.016a | <1 | <1 |
| 56 | Acetovanillone | 2610 | 2620 | 867 | 54.2 | 6.52 | 0.117±0.014 | - | <1 | - |
| 57 | Myristic acid | 2663 | 2670 | 850 | 55.80 | 5.30 | 0.288±0.022a | 0.571±0.054b | <1 | <1 |
| 58 | Palmitic acid | 2864 | 2890 | 863 | 59.67 | 6.00 | 1.644±0.155a | 1.784±0.157a | - | - |
| 59 | 4-hydroxyphenethyl alcohol | 2983 | 2985 | 790 | 61.27 | 4.28 | 0.100±0.007a | 0.163±0.008b | - | - |
| 60 | 2-octanone | 1288 | 1319 | 825 | 12.07 | 6.94 | 0.098±0.014a | 0.075±0.003a | 7 | 5 |
| 61 | 3-methyl-1-pentanol | 1335 | 1309 | 839 | 14.33 | 3.52 | 0.028±0.002a | 0.025±0.003a | <1 | <1 |
| 62 | 5-methylfuranaldehyde | 1558 | 1570 | 872 | 23.53 | 3.64 | 0.027±0.001 | - | <1 | - |
| 63 | γ-butyrolactone | 1592 | 1640 | 914 | 25.13 | 5.28 | 0.093±0.007a | 0.055±0.004b | <1 | <1 |
| 64 | 1-nonanol | 1661 | 1664 | 843 | 27.40 | 6.82 | 0.004±0.000 | - | <1 | - |
| 65 | 1,3-propanediol | 1806 | 1789 | 760 | 32.47 | 2.62 | 0.012±0.001a | 0.155±0.012b | - | - |
| 66 | Phenol | 1982 | 2011 | 871 | 39.00 | 2.86 | 0.052±0.001a | 0.042±0.002b | <1 | <1 |
| 67 | 2-methylpyrazine | 1257 | 1267 | 887 | 11.00 | 3.54 | - | 0.008±0.000 | - | <1 |
| 68 | 2-methylbutyl acetate | 1104 | 1128 | 785 | 5.53 | 5.68 | - | 0.159±0.009 | - | 14 |
| 69 | Dehydromevalonolactone | 1963 | 1967 | 841 | 38.47 | 3.84 | 0.017±0.002a | 0.014±0.000a | - | - |
| 70 | 9-decenoic acid | 2319 | 2348 | 811 | 48.20 | 3.74 | - | 0.028±0.002 | - | - |
| 71 | Pyridine | 1170 | 1181 | 864 | 7.93 | 3.10 | 0.010±0.001a | 0.005±0.000b | - | - |
| 72 | m-xylene | 1152 | 1146 | 748 | 7.27 | 5.62 | - | 0.037±0.003 | - | <1 |
| 73 | Ethyl hexanoate | 1238 | 1241 | 831 | 10.33 | 8.36 | 0.092±0.004a | 0.114±0.009b | 18 | 23 |
| 74 | Acetic acid | 1494 | 1473 | 769 | 20.60 | 2.34 | 1.803±0.089a | 0.015±0.002b | <1 | <1 |
| 75 | Ethyl palmitate | 2319 | 2274 | 814 | 48.20 | 8.22 | - | 0.026±0.001 | - | <1 |
| 76 | Phenyl acetaldehyde | 1618 | 1640 | 850 | 26.07 | 4.22 | - | 0.292±0.008 | - | 292 |
| 77 | Styrene | 1242 | 1257 | 923 | 10.47 | 4.72 | - | 0.012±0.002 | - | <1 |
| 78 | 2-phenyl-2-butenal | 1892 | 1922 | 881 | 36.33 | 0.90 | 0.035±0.002a | 0.015±0.000b | - | - |
| 79 | Trioxymethylene | 1156 | 1167 | 789 | 7.40 | 2.76 | 0.078±0.004a | 0.084±0.003a | - | - |
| 80 | Ethyl pyruvate | 1272 | 1234 | 839 | 11.53 | 3.26 | 0.050±0.001a | 0.071±0.003b | <1 | <1 |
| 81 | N,N-dimethylacetamide | 1387 | 1414 | 847 | 17.00 | 3.36 | 0.010±0.001a | 0.005±0.000b | - | -- |
| 82 | 2,3-butanediol | 1572 | 1581 | 834 | 24.20 | 4.62 | 0.598±0.014a | 0.313±0.027b | -- | - |
| 83 | Diethyl malonate | 1575 | 1572 | 823 | 24.33 | 4.92 | 0.008±0.000a | 0.011±0.001a | - | - |
| 84 | Ethyl levulinate | 1592 | 1607 | 809 | 25.13 | 4.50 | 0.040±0.002a | 0.019±0.002b | <1 | <1 |
| 85 | n-ethylactamide | 1627 | 1608 | 783 | 26.33 | 2.88 | 0.130±0.016a | 0.188±0.011a | - | - |
| 86 | Phenethyl formate | 1774 | 1768 | 835 | 31.27 | 5.44 | 0.017±0.002a | 0.027±0.002b | <1 | <1 |
| 87 | Ethyl phenylacetate | 1781 | 1771 | 851 | 31.53 | 6.96 | 0.025±0.002a | 0.022±0.002a | <1 | <1 |
| 88 | Ethyl nicotinate | 1803 | 1782 | 850 | 32.33 | 5.92 | 0.157±0.003a | 0.175±0.008a | <1 | <1 |
| 89 | 1,2,3-trimethoxybenzene | 1935 | 1955 | 845 | 37.67 | 6.38 | 0.011±0.002a | 0.008±0.001a | - | - |
| 90 | β-ethylphenylethanol | 1944 | 1950 | 811 | 37.93 | 5.38 | 0.010±0.001a | 0.007±0.000a | - | - |
| 91 | 4-ethyl-2-methoxyphenol | 2001 | 2032 | 871 | 39.53 | 5.44 | 0.009±0.001a | 0.013±0.001a | 1 | 1 |
| 92 | Diethyl malate | 2023 | 2060 | 839 | 40.20 | 4.90 | 0.063±0.002a | 0.041±0.002a | <1 | <1 |
| 93 | 2-Piperidone | 2096 | 2060 | 873 | 42.47 | 3.42 | - | 0.051±0.003 | - | - |
| 94 | 2-vinyl-4-methoxyphenol | 2175 | 2180 | 828 | 44.47 | 4.26 | 0.154±0.003a | 0.194±0.017a | - | - |
| 95 | 2,4,5-trimethoxybenzadehye | 2216 | 2233 | 676 | 45.53 | 5.58 | 0.050±0.004a | 0.208±0.021b | - | - |
| 96 | Dimethyl phthalate | 2265 | 2276 | 856 | 46.87 | 4.90 | 0.019±0.002a | 0.071±0.005b | - | - |
| 97 | Levulinic acid | 2300 | 2312 | 839 | 47.80 | 2.64 | 0.402±0.012 | - | - | - |
| 98 | Monoethyl succinate | 2370 | 2368 | 808 | 49.27 | 4.78 | 3.124±0.261a | 4.145±0.304a | <1 | <1 |
| 99 | Phenylacetic acid | 2535 | 2568 | 887 | 52.73 | 2.84 | 0.421±0.039a | 0.354±0.059a | <1 | <1 |
| 100 | Vanillin | 2535 | 2568 | 876 | 52.73 | 3.34 | 0.490±0.020a | 0.797±0.023b | 8 | 13 |
| 101 | N-(2-phenylethyl) acetamide | 2557 | 2580 | 840 | 53.13 | 3.78 | 0.090±0.003a | 0.179±0.013b | - | - |
| 102 | Dibutylphthalate | 2659 | 2680 | 840 | 55.67 | 8.36 | 0.151±0.012a | 0.081±0.005b | - | - |
| 103 | Syringaldehyde | 2897 | 2930 | 898 | 60.07 | 3.50 | 0.080±0.009a | 0.045±0.004b | <1 | <1 |
| 104 | Hexanal | 1054 | 1081 | 777 | 4.33 | 4.08 | 2.024±0.145 | - | 3 | - |
| 105 | Isovaleramide | 1895 | 1903 | 775 | 36.47 | 2.96 | 0.014±0.002a | 0.014±0.001a | - | - |
| 106 | 3,3-dimethyl-2-butanol | 1100 | 1114 | 718 | 5.40 | 6.20 | 0.062±0.001a | 0.084±0.003b | - | - |
| 107 | Propylene glycol | 1581 | 1589 | 850 | 24.60 | 3.52 | - | 0.147±0.014 | - | <1 |
| 108 | 2-pyrrolidone | 2014 | 2037 | 856 | 39.93 | 3.12 | - | 0.029±0.002 | - | - |
| 109 | Ethyl vanillate | 2610 | 2615 | 848 | 54.20 | 4.12 | 0.100±0.002a | 0.136±0.005b | <1 | <1 |
| 110 | Sugar lactone | 2175 | 2186 | 828 | 44.47 | 5.94 | 0.030±0.001 | - | 6 | - |
| 111 | N,N-dimethylformamide | 1320 | 1319 | 801 | 13.53 | 5.94 | 0.017±0.001 | - | - | - |

^a^ The linear retention indices determined by the experiment.

^b^ The linear retention indices reported from the literature.

^c^ The retention time of volatile compounds on the 1D column of GC × GC-qMS.

^d^ The retention time of volatile compounds on the 2D column of GC × GC-qMS.

^e^ Values with different superscript roman letters (a–c) in the same row are significantly different according to the Duncan test (*p* < 0.05).

^f^ Not detected in sample.
